# Nascentome Analysis Uncovers Futile Protein Synthesis in *Escherichia coli*

**DOI:** 10.1371/journal.pone.0028413

**Published:** 2011-12-05

**Authors:** Koreaki Ito, Yuhei Chadani, Kenta Nakamori, Shinobu Chiba, Yoshinori Akiyama, Tatsuhiko Abo

**Affiliations:** 1 Faculty of Life Sciences, Kyoto Sangyo University, Kyoto, Japan; 2 Graduate School of Natural Science and Technology, Okayama University, Okayama, Japan; 3 Institute for Virus Research, Kyoto University, Kyoto, Japan; University of Crete, Greece

## Abstract

Although co-translational biological processes attract much attention, no general and easy method has been available to detect cellular nascent polypeptide chains, which we propose to call collectively a “nascentome.” We developed a method to selectively detect polypeptide portions of cellular polypeptidyl-tRNAs and used it to study the generality of the quality control reactions that rescue dead-end translation complexes. To detect nascent polypeptides, having their growing ends covalently attached to a tRNA, cellular extracts are separated by SDS-PAGE in two dimensions, first with the peptidyl-tRNA ester bonds preserved and subsequently after their in-gel cleavage. Pulse-labeled nascent polypeptides of *Escherichia coli* form a characteristic line below the main diagonal line, because each of them had contained a tRNA of nearly uniform size in the first-dimension electrophoresis but not in the second-dimension. The detection of nascent polypeptides, separately from any translation-completed polypeptides or degradation products thereof, allows us to follow their fates to gain deeper insights into protein biogenesis and quality control pathways. It was revealed that polypeptidyl-tRNAs were significantly stabilized in *E. coli* upon dysfunction of the tmRNA-ArfA ribosome-rescuing system, whose function had only been studied previously using model constructs. Our results suggest that *E. coli* cells are intrinsically producing aberrant translation products, which are normally eliminated by the ribosome-rescuing mechanisms.

## Introduction

Translation of genetic information into protein is supported by multi-faceted quality control mechanisms [1], in which the core of the processes involves several major players of translation, including a messenger RNA, the ribosome, polypeptidyl and aminoacyl-tRNAs, as well as initiation, elongation and termination factors. In spite of the obligatory nature of ribosome-tethered polypeptidyl-tRNAs in translation, their *in vivo* behaviors have not been studied extensively, as conventional views have assumed that they exist only for insignificant time and are devoid of any function.

However, the rate of polypeptide chain elongation along an mRNA is not necessarily uniform [2], [3]. Such variations in elongation speed could arise not only at the decoding and peptide bond formation steps [4], [5], [6], [7] but also at the subsequent steps, in which newly synthesized polypeptide segments are inspected by the exit tunnel of the ribosome to receive elongation control "retrospectively". The known examples of the latter category of regulation involve ribosome-stalling amino acid sequences in the nascent chains [8], [9], [10], [11], [12], [13], [14], [15]. In these cases, certain species of polypeptidyl-tRNAs can exist for extended lengths of time during translation. Regulatory nascent chains in bacteria, such as SecM, MifM, ErmCL and TnaC, contain well characterized amino acid sequences that interact with the ribosomal interior components and arrest the elongation step of translation in manners regulated by specific physiological conditions; the elongation arrest in turn serves as a novel mechanism of gene regulation [9], [10], [11], [13], [14]. More generally, elongation speed of translation could be fine-tuned in such a way to facilitate certain co-translational events, such as subcellular targeting of the nascent chain or of the messenger RNA [16], [17], as well as folding and assembly of gene products [3], [18]. Intriguing possibilities are conceivable that the facilitated events in turn feed-back regulate the elongation speed [13].

The ribosome also stalls upon aberrant events in translation, for instance when it encounters the end of messenger RNA without an in-frame stop codon (non-stop mRNA) [19], [20]. Such a class of polypeptidyl-tRNA-bearing ribosomes must be rescued to allow them to dissociate from the dead-end translation complexes and to function again. It was shown recently that the ribosomes stalled on non-stop mRNAs are rescued by dual mechanisms in *Escherichia coli*, tmRNA-mediated trans-translation and ArfA-mediated peptidyl-tRNA hydrolysis [21]. However, these studies have used model systems to produce non-stop messages and, therefore, the generality and extent of the occurrence of these modes of quality control in the expression of native chromosomal genes have not been established.

Undoubtedly, specific detection of nascent polypeptides will facilitate our understanding of a number of emerging issues in translation and co-translational protein maturation. Since the carboxyl ester bond between an amino acid and its cognate tRNA is generally labile at elevated pH and temperature [22], [23], [24], a large fraction of nascent polypeptidyl-tRNA molecules may lose the tRNA moieties during most formats of SDS-PAGE separation. However, only limited information is available about the stability of polypeptidyl-tRNAs [22] and attempts have been rare to detect polypeptidyl-tRNAs in living cells [25], [26]. Here, we examined stabilities of polypeptidyl-tRNAs having different aminoacyl-tRNA ester linkages. Based on this survey, we developed a simple two-dimensional electrophoretic method to detect polypeptide portions of cellular polypeptidyl-tRNAs separately from regular polypeptides, which lack any tRNA moieties. Using this method we have revealed that *E. coli* cells experience unexpectedly frequent incidence of futile protein synthesis, which is normally masked by the ribosome rescuing factors.

## Results

### Non-uniform Stability of Polypeptidyl-tRNAs

In our attempts to detect cellular nascent polypeptides, we intended to make use of the fact that peptidyl-tRNA ester bonds can be preserved during separation under neutral pH conditions by the Bis-Tris NuPAGE system (Invitrogen) [27], [28]. By contrast, they are often hydrolyzed during separation by conventional Laemmli system of SDS-PAGE [27]. Our strategy was to separate *E. coli* proteins by NuPAGE and then attempt in-gel cleavage of the ester bonds by incubating excised gel lanes at high pH and at high temperature. By subjecting thus treated first dimension gel lane to the second dimension electrophoresis, we will be able to separate nascent polypeptides from translation-completed polypeptide chains.

**Figure 1:**
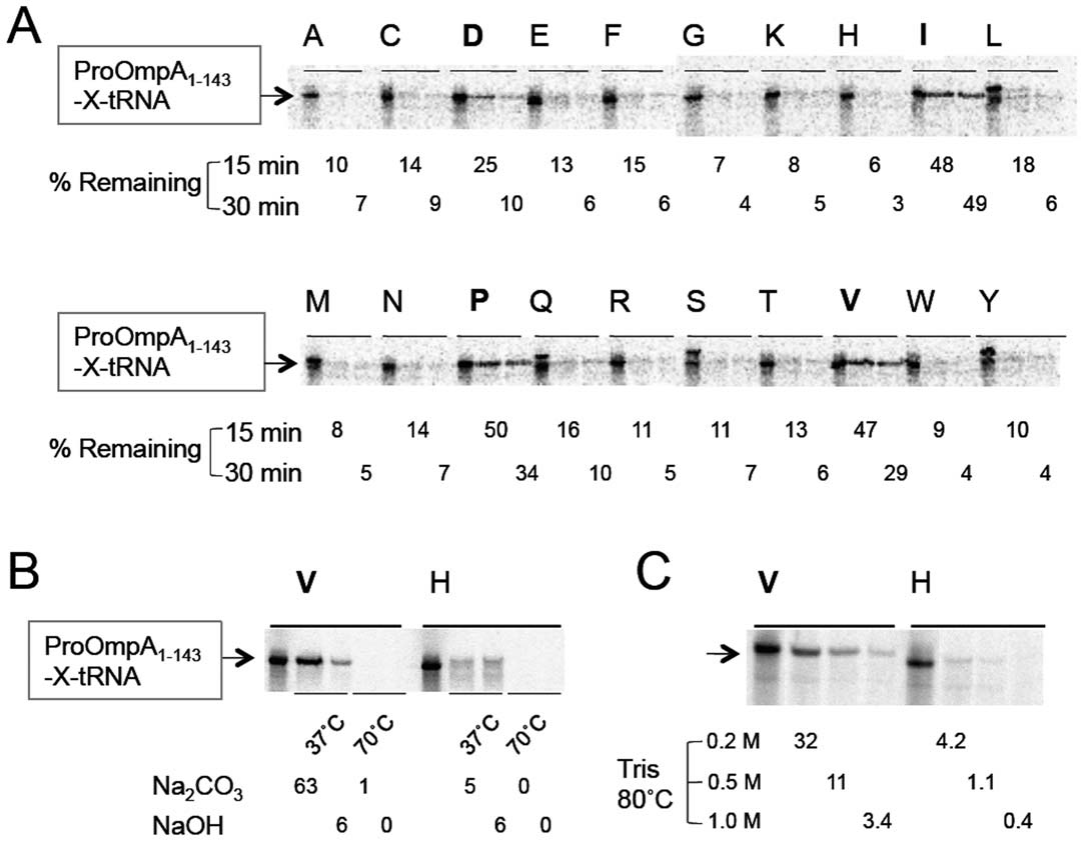
Non-uniform stability of polypeptidyl-tRNA ester bonds. (A) *In vitro* translation was directed by a truncated proOmpA_1-143_ (Met1 to Thr143) message that was followed by a codon for one of 20 amino acids [27]. Reaction was allowed at 37°C for 30 min in the presence of [^35^S]methionine. Macromolecules were precipitated with trichloroacetic acid (final concentration, 5%) and the precipitates were washed twice with acetone and dissolved in 2 x Laemmli sample buffer [32] (see Materials and Methods for the composition) that had been treated with RNASecure (Ambion), at room temperature followed by incubation at 37°C for 5 min. Samples were then divided into 3 portions. The first sample was untreated. The second and the third samples were mixed with an equal volume of 0.4 M Tris-base and incubated at 70°C for 15 and 30 min, respectively. They were separated by NuPAGE to visualize ^35^S-labeled proOmpA_1-143_-X-tRNA bands with phosphor imaging. Numbers indicate intensities of the bands, after incubation with Tris-base, relative (%) to that of the untreated sample. (B) Stability of proOmpA_1-143_-Val-tRNA and proOmpA_1-143_-His-tRNA was examined as described in (A) except that protein precipitates were dissolved in 1 x sample buffer, to which 1/10 volume of either 2 M Na_2_CO_3_ or 2 N NaOH was added and incubated at 37°C or 70°C for 15 min as indicated. (C) Stability of proOmpA_1-143_-Val-tRNA and proOmpA_1-143_-His-tRNA was examined as described in (A) except that samples were mixed with an equal volume of either 0.4 M, 1.0 M or 2.0 M Tirs-base and incubated at 80°C for 20 min as indicated.

While earlier studies described stability of different aminoacyl-tRNA species [23], [24], [29], [30], only limited knowledge is available about the stability of polypeptidyl-tRNAs [22]. As described in Supporting Information, our preliminary results suggested that carboxyl ester bonds of some polypeptidyl-tRNA species were highly refractory to hydrolysis under moderately alkaline pH at 70°C (Figures S1 and S2). To determine which amino acids confer the hydrolysis-resistance when present at the C-terminus of polypeptidyl-tRNA, we used an *in vitro* translation system [31] to prepare a series of nascent proOmpA'-tRNAs having the N-terminal 143 amino acids followed by one of the 20 different amino acids (Figure 1). Translation products in two-fold concentrated SDS sample buffer [32] was mixed with 0.4 M tris(hydroxymethyl)aminomethane (Tris-base) to make final pH of ∼8.8 and incubated at 70°C. Detection of ProOmpA-tRNAs by NuPAGE electrophoresis revealed that molecules that contained Ile, Pro or Val before tRNA were more refractory to hydrolysis than those contained other amino acids at this position (Figure 1A). Asp conferred similar but less pronounced hydrolysis-resistance (Figure 1A). We then found that incubation at 70°C with stronger alkali (0.2 M Na_2_CO_3_ or 0.2 N NaOH) resulted in complete hydrolysis of the peptidyl-valyl-tRNA bond (Figure 1B). However, it turned out that we could not use these strongly alkaline conditions for in-gel hydrolysis, because proteins became immobilized due presumably to the formation of some covalent bonds with acrylamide components of the gel [33], [34].

### Two Dimensional SDS-PAGE to Detect Cellular “Nascentome”

Therefore, we had to find out a condition that allows nearly complete cleavage of the resistant class of ester bonds but keeps gel-separated proteins from adversely interacting with the gel components. After a number of trials, we came to the conclusion that incubation with 1 M Tris-base at 80°C for 20 min fulfills this requirement, leading to effective, if not 100%, cleavage of the polypeptidyl-valyl-tRNA (Figure 1C). The amino group of Tris-base probably enhances the ester bond cleavage by aminolysis [30], [35], while it appears to compete with proteins in the gel in interacting with gel-derived acrylamide components.

**Figure 2:**
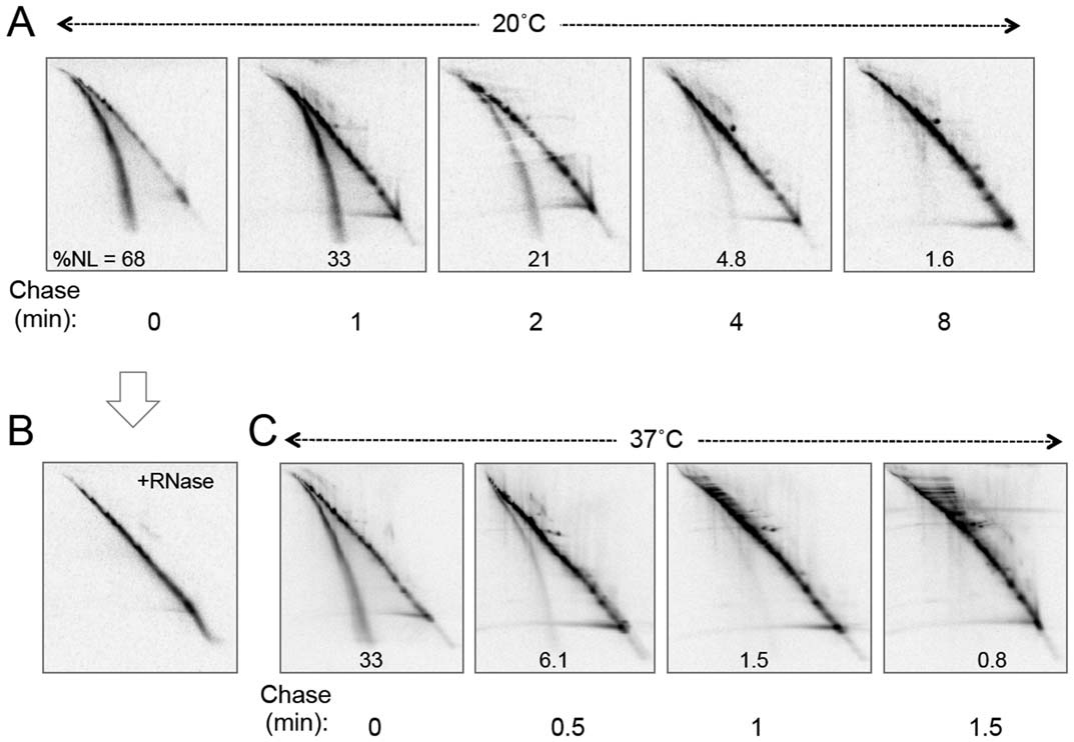
Detection of the *E. coli* nascentome by pulse-chase labeling and two-dimensional separation. (A and C) *E. coli* strain W3110 was grown at 20°C (A) or 37°C (C) and pulse-labeled for 0.5 min with [^35^S]methionine and chased with unlabeled methionine for the indicated lengths of time. Samples were prepared as described in Materials and Methods and subjected to separation by the nascentome two-dimensional electrophoresis. Radioactive materials were visualized by phosphor imaging. The upper (main diagonal) line represents translation-completed polypeptide and the lower line (nascent line) represents polypeptide portions of polypeptidyl-tRNAs originally present in the cell. The value of %NL (proportion of radioactivities on the nascent line in the total radioactivities displayed on the gel) is shown in each gel panel. (B) A sample identical to that of zero-chase in (A) was prepared without using RNASecure and incubated with 0.1 mg/ml of RNase A at 30°C for 20 min before electrophoresis.

For two-dimensional separation, a lane of the first dimension Nu-PAGE gel was cut out and incubated under the above conditions, followed by its placement on the top of the second dimension gel (Materials and Methods for details). We pulse-labeled *E. coli* cells with [^35^S]methionine for 0.5 min at 20°C and immediately denatured and precipitated macromolecules with trichloroacetic acid. The precipitates were then dissolved in SDS. A portion of the sample was treated with RNase A to hydrolyze any tRNA moiety of the nascent chains. As shown in Figure 2A (the first panel), the sample without the RNase treatment gave prominent two lines of radioactive materials on the second dimension gel. Remarkably, the RNase-treatment eliminated the lower line and increased the intensity of the upper, main diagonal line (Figure 2B), indicating that the materials on the lower line was converted into materials that aligned on the latter. These results substantiate the notion that the lower line was formed by molecules that originally contained RNA. Our experiences with SecM [27] and MifM [36] indicate that the apparent difference in the electrophoretic mobilities between a polypeptidyl-tRNA and its polypeptide component corresponds to about 18 kDa. This value can well explain the migration difference between the upper line and the lower line in the second dimension electrophoresis. Because tRNAs are nearly homogenous in their sizes, polypeptidyl-tRNAs lose a fixed molecular mass in the second dimension and form a continuous line below the main diagonal line. Thus, we designate the lower line observed after the two-dimensional separation as a “nascent line” (NL in Figure 2A), because it is formed by a collection of polypeptides that have been linked to a tRNA *in vivo*. We conclude that the nascent line represents the polypeptide portions of nascent polypeptidyl-tRNAs in the cell and propose a portmanteau word “nascentome” to refer to the entire set of nascent polypeptides expressed by a genome at a given time. A characteristic feature of nascentome is that a major class of its members will only exist transiently and will be overwhelmingly diverse and complex as compared with the distinct proteome members. However, we expect that a nascentome will also contain another class of members that are distinct, more stable and produced by elongation stalling.

### Polypeptidyl-tRNAs as Intermediates in Translation

When *E. coli* cells were pulse-labeled at 20°C with [^35^S]methionine for 0.5 min, nearly 70% of pulse-labeled proteins were found on the nascent line and, hence, it was likely that they were still in the process of elongation. As a global indication of the incompleteness of polypeptide chains, we use the value of %NL, the proportion of radioactivities associated with the nascent line in the total radioactivities displayed on a gel. During the chase, the %NL values decreased gradually taking about 4 minutes to reach a steady state value of <1% (Figure 2A). As expected, incomplete chains were converted into regular proteins much more rapidly at 37°C, at which elongation speed is expected to be higher than at 20°C; the %NL value decreased to the background, steady state value in ∼1.5 min at 37°C (Figure 2C), with an apparent global half life of ∼14 sec.

We did not detect any appreciable radioactive spot on the nascent line after chase for 4 min at 20°C or 1.5 min at 37°C, indicating that *E. coli* does not possess any highly abundant and long-lasting nascent polypeptide under normal growth conditions. This seems reasonable because (i) regulatory arrest peptides may not be expressed abundantly and (ii) in many cases their elongation arrest will only be effective under specific physiological conditions. For instance, the elongation arrest of SecM is rapidly released unless the activity of the Sec protein export machinery is compromised [9], [37], [38]. In fact, we were able to detect SecM on the nascent line when it lacked a functional signal sequence and was overproduced from a plasmid (Supporting Information, Figure S3). This SecM variant [(ΔLGLPA)SecM], which undergoes constitutive elongation arrest [38], remained on the nascent line even after a chase or upon visualization with silver- or immuno-staining (Supporting Information; Figure S3).

### Futile Protein Synthesis Revealed by Detection of Aberrant Polypeptidyl-tRNAs in *E. coli* Defective in the tmRNA-ArfA Ribosome-Rescuing Mechanisms

The rapid elimination of cellular polypeptidyl-tRNAs does not necessarily indicate that all of them are productive intermediates in translation. Some of them might be eliminated because they were produced by translation complexes that face a trouble. For instance nucleolytic cleavage of mRNA or premature termination of transcription can lead to the production of aberrant mRNAs (non-stop mRNAs) that lack any in-frame stop codon. The ribosome will then stall at the 3′ end, with its A-site kept vacant. Such aberrant stalling of the ribosome, accompanied by a P-site-tethered peptidyl-tRNA, can be deleterious because the active ribosomes become sequestered, a situation that must be rescued by cellular quality control mechanisms [20], [21], [39], [40], [41], [42]. However, studies on this subject have generally used model non-stop mRNA constructs and, therefore, it has been no direct data showing how extensively aberrant ribosome stalling takes place *in vivo*. Our electrophoretic system, in conjunction with the availability of the *E. coli* mutant defective in the ribosome-rescuing mechanisms [21], offers an excellent means to answer this question.

It is believed that transfer-messenger RNA (tmRNA)-mediated trans-translation rescues a stalled ribosome by allowing translation to continue on its own message and then to terminate on its own stop codon [41], [43]. However, single disruption of *ssrA* (encoding tmRNA) does not hamper growth of *E. coli* [44]. Recently, Chadani, et al [21] identified an alternative ribosome rescue-factor, ArfA, through a search for a mutation that is synthetically lethal with deletion of the *ssrA* gene. We examined whether polypeptidyl-tRNAs accumulate for prolonged time as a consequence of tmRNA and ArfA double depletion.

**Figure 3:**
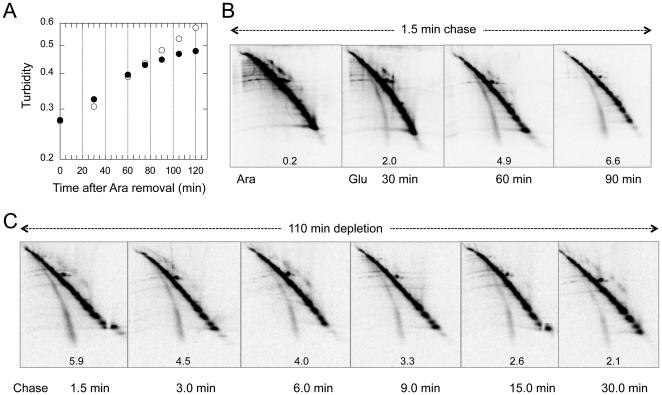
Stabilization of aberrant polypeptidyl-tRNAs upon dysfunction of the tmRNA-ArfA ribosome-rescuing system. (A) CH111 (Δ*ssrA*Δ*arfA*/pBAD24-*ssrA^+^*) cells grown at 37°C in the presence of arabinose were harvested by centrifugation, washed and resuspended in the medium containing arabinose (open circles) or glucose (solid circles). Growth curves were recorded by absorbance measurement at 660 nm. (B) Portions of the same cultures as shown in (A) were sampled for pulse-labeling and nascentome two-dimensional separation. Cells in the presence of arabinose (Ara) as well as those at indicated time points after the arabinose to glucose (Glu) medium change were pulse-labeled for 0.5 min followed by chase for 1.5 min. Radioactive samples were prepared and separated in two-dimensions as described in Figure 2. The %NL value is given in each gel panel. (C) CH111 cells at 110 min after arabinose to glucose medium change were pulse-labeled for 0.5 min and chased for the indicated lengths of time for electrophoretic visualization of radioactive polypeptidyl-tRNAs. The %NL value is given in each gel panel.

A Δ*ssrA* Δ*arfA* double disruption mutant that was complemented with arabinose promoter-controlled *ssrA* ^+^ on a plasmid requires the inducer arabinose for growth (Figure 3A). In the presence of arabinose, the mutant cells exhibited a normal pattern of pulse-chase labeling; the value of %NL decreased to <1 within 1.5 min at 37°C (Figure 3B, Ara). Upon arabinose to glucose change in the carbon source, cell growth continued for about 80 min with normal rate and then declined gradually (Figure 3A). At different time points after the medium change, cells were pulse-labeled for 0.5 min and then chased for 1.5 min. At 30 min after the medium change, when cell growth was not yet affected, a significant fraction of pulse-labeled materials remained on the nascent line even after the 1.5 min chase (%NL = 2; Figure 3B, Glu 30 min). The %NL value increased to 4.9 at 60 min and to 6.6 at 90 min. Because stabilization of the polypeptidyl-tRNAs was observed as early as 30 min, when neither cell growth nor bulk protein synthesis was affected, this phenotype was not a non-specific consequence of general inhibition of protein synthesis. Instead, it could well be a causative of the later cessation of protein synthesis and growth.

To examine how pronouncedly the double depletion of ArfA and tmRNA stabilized the fraction of polypeptidyl-tRNAs, we carried out longer chases at 110 min after the medium change (Figure 3C). The nascent line was visible even after a chase for as long as 30 min (Figure 3C), although the %NL values decreased gradually from the initial value of ∼6 to ∼2 at 30 min. The slow decrease during the chase may have been due to still another rescue factor [45], residual occurrence of trans-translation, or proteolytic degradation.

Cells with a single Δ*arfA* mutation did not exhibit any stabilization of polypeptidyl-tRNAs (Figure S4, the right panel) but those with a single Δ*ssrA* mutation appeared to have a slightly stabilized fraction of polypeptidyl-tRNAs (Supporting Information, Figure S4, the middle panel). The stabilization of polypeptidyl-tRNAs by the single Δ*ssrA* mutation was much less pronounced than that observed with the doubly depleted cells. The weak defect observed with the Δ*ssrA* cells is consistent with the argument [21], [46] that trans-translation plays a major role in rescuing the stalled ribosomes, while ArfA has a fail-safe function in this two-member rescue system. We have thus revealed that in the simultaneous absence of tmRNA and ArfA, a few percent of newly synthesized polypeptides remain in the states of polypeptidyl-tRNAs for prolonged time. ArfA tightly associates with the ribosomal large subunit and somehow induces hydrolysis of peptidyl-tRNAs tethered to the stalled ribosome [21]. Our present results indicate that the native chromosomal genes of *E. coli* indeed undergo aberrant events of translation, leading to the production of a class of stable polypeptidyl-tRNAs, which become observable in the simultaneous absence of tmRNA and ArfA.

## Discussion

Every protein molecule of the cell has experienced nascent states, in which growing polypeptide chain had been linked covalently to a tRNA to receive elongation reaction at the peptidyl-transferase active center of the ribosome. An increasing body of attention is being directed in recent years to the elongation step of translation, as it sometimes receives programmed and environment-responding regulation. On the other hand, to maintain cellular capability of protein synthesis, the ribosomes should not be kept in the elongation complex too long even in a situation, in which they have encountered a difficulty in terminating translation. Although living cells are equipped with mechanisms that rescue aberrant translation complexes on non-stop mRNAs, the extent of occurrence of the rescuing reaction has been unknown. One of the reasons for this lack of information is that we did not have appropriate method to directly detect nascent polypeptides of the cell.

Our electrophoretic method rectifies this gap and provides us with a new direction of approaches to the biology of nascent polypeptides. The two-dimensional separation is based on a simple and universal principle; instability of the carboxyl-ester bond between the last amino acid of a nascent polypeptide and adenosine moiety of tRNA, making our method applicable to analysis of nascent chains in cells of any organism. During the course of this study, we encountered an unexpected fact that some polypeptidyl-tRNAs are quite robust such that their hydrolysis required harsh conditions of high temperature and strongly alkaline pH. The amino acids valine, isoleucine and proline identified in this study as making a hydrolysis-resistant ester bond when present at the carboxyl end of a nascent polypeptide do not exactly coincide with the relatively stable class of aminoacyl-tRNAs studied earlier [23], in which prolyl-tRNA was assigned as unstable. The underlying mechanism for this discrepancy is unknown but it may be pointed out that polypeptidyl-tRNAs are generally more stable than free aminoacyl-tRNAs [22] and that peptidyl-prolyl bond can undergo *cis*-*trans* isomerization [47].

Our optimization experiments show that incubation of the first dimension gel lane with 1 M Tris-base at 80°C is appropriate for efficient cleavage of the ester bonds of polypeptidyl-tRNAs without causing anomalous interactions of the molecules with the gel components, which proved to take place under high-pH–high-temperature conditions. Presumably, the amino group of Tris-base not only facilitates hydrolysis/aminolysis of the ester bonds [30], [35] but also competes with amino groups of the polypeptides in interacting with the presumed gel-derived materials [33], [34]. Now molecules that originally bared a tRNA have lost the tRNA and become to migrate faster in the second dimension electrophoresis.

The polypeptide portions of cellular polypeptidyl-tRNAs can be seen as a “nascent line” below the main diagonal line that is formed by regular, completed chains of cellular proteins. In conventional methods, nascent chains of a particular protein are expected to be detectable as polypeptides shorter than the elongation-completed chain. However, such shorter fragments can arise not only as a nascent element in biosynthesis but also as a degradation product of the completed chain, making it difficult to detect specifically the former. In our method, any polypeptide that is present on the nascent line after the two-dimensional separation can be regarded with high degree of confidence to have been in the forms of polypeptidyl-tRNAs *in vivo*. Separation of cellular proteins into those bearing a covalently attached tRNA and those that have completed the translation processes enables us to grasp the overall picture of protein biogenesis by pulse-chase labeling. The easy and unambiguous detection of nascent chains makes the concept of “nascentome” amenable to experimental analysis and will open up an avenue to studying aspects of quality control of translation complexes as well as co-translational maturation and modification of proteins.

Application of our electrophoretic system to the analysis of polypeptidyl-tRNAs of the *E. coli* mutant defective in both tmRNA and ArfA has yielded the results that indicate that a significant fraction of newly synthesized polypeptidyl-tRNAs are in non-productive states, which are subject to quality control by the ribosome-rescuing system. As tmRNA and ArfA cooperate to rescue aberrant states of the ribosome due typically to the lack of normal stop codon in the mRNA, the accumulated polypeptidyl-tRNAs should represent incomplete translation products encoded by non-stop mRNAs. Our results indicate that the incidence of aberrant translation is quite frequent in *E. coli* cells although it is usually masked by the ribosome-rescuing factors that either lead to tmRNA-mediated trans-translation followed by termination or ArfA-mediated hydrolysis of polypeptidyl-tRNAs. The simultaneous absence of the trans-translation and the hydrolysis mechanisms leads to the significantly increased accumulation of polypeptidyl-tRNAs; as much as some 2–4% of newly synthesized proteins are stabilized in the double mutant strain.

A previous estimation of trans-translation on the basis of tagging with an engineered tmRNA showed that approximately 0.4% of proteins terminates with tmRNA-mediated tagging in normally growing *E. coli* cells [48]. However, this value should be regarded as showing the lower limit with respect to the actual occurrence of the targets of the ribosome rescue-system, because ArfA might have channeled a significant fraction of aberrant translation complexes to the hydrolysis pathway of rescue. Recent studies have revealed that ArfA itself is produced physiologically from the non-stop message in a manner destabilized by the trans-translation-mediated proteolysis [46], [49]. Therefore, the use of the proteolysis-refractory tag sequence might have stabilized ArfA in the previous estimation of *in vivo* occurrence of trans-translation [48], leading to up-regulated operation of the hydrolysis pathway and to the decreased general tagging by trans-translation.

It should be noted that the programmed and productive ribosome stalling, in SecM for instance, is not a target of the rescue mechanisms; this mode of robust stalling produces a state of the ribosome, in which A-site is occupied by the codon-specified aminoacyl-tRNA that precludes an access by tmRNA [27], [50]. By contrast, ribosome stalling at the end of non-stop mRNA produces the A-site-vacant state of the ribosome, which becomes a platform for recruitment of various quality control factors [21], [40], [51], [52]. Identification of the polypeptidyl-tRNAs accumulated in the absence of the ribosome-rescue system will be of prime importance in the immediate future. Whereas some of them could be produced as a consequence of an aberrant or stressful event, others might be produced in a programmed fashion, as is the case for ArfA itself [46], [49]. The steady state occurrence of futile protein synthesis found in *E. coli* provides an additional example that nature's design of gene expression is not to attempt perfect jobs. Instead, living organisms take the strategy of continuously correcting the erroneous outcomes produced at non-negligible frequencies.

A majority of the polypeptidyl-tRNAs detected in the wild-type cells by our pulse-labeling experiments appears to be in the process of elongation, in which they are converted into the completed chains during the chase. The %NL values decrease with a rough half time of ∼14 sec at 37°C (Figure 2C) and ∼65 sec at 20°C (Figure 2B). If elongation of nascent polypeptide occurs at a rate of 12–21 amino acids per second at 37°C [4], the observed half life (∼14 sec) of the nascentome intensity corresponds to a time required for polymerizing 168–252 amino acids and agrees reasonably well with the time required for completion of average-sized *E. coli* proteins.

However, it is likely that this kind of global analysis overlooks proteins expressed at low levels and any fine variations in the elongation speeds of individual proteins. Profiling the elongation completion of individual proteins will be possible by using an N-terminal affinity tag in combination with the nascentome electrophoresis. Such analysis is expected to reveal elongation pausing within a coding region of certain class of proteins as distinct patterns of the nascent line upon pulse-labeling. While the methodology of ribosome profiling [2] is powerful in obtaining *in vivo* snapshots of ongoing translation at the nucleotide-level resolution, the time-wise resolution of our method, which could follow dynamic changes of a nascent translation product, could play complementary roles. Although our results show that *E. coli* does not possess any abundantly expressed protein that undergoes constitutive elongation arrest, new regulatory nascent chains could still be identified by our electrophoretic technique. Nascentome analysis will enable us to address whether the rate of polypeptide chain elongation can be affected by the behaviors of the nascent chain. Interesting problems we can address in this direction include whether conditions disturbing protein homeostasis, such as depletion or overproduction of molecular chaperones, targeting factors or partners of molecular assemblies affects elongation speed of the target nascent polypeptide.

## Materials and Methods

### Escherichia coli Strains

Strain W3110 was used as an *E. coli* K12 wild-type strain. TA331 (W3110 Δ*ssrA*::FRT) and CH101 (W3110 Δ*arfA*::FRT) were described previously [21]. CH111 was a derivative of W3110, carrying the Δ*arfA*::FRT and the Δ*ssrA*::FRT mutations on the chromosome as well as plasmid pBAD24-*ssrA* ^+^ [21].

### Media and Pulse-Chase Labeling

For pulse-labeling experiments we used M9 medium [53] (with omission of CaCl_2_) supplemented with amino acids (20 µg/ml each, other than methionine and cysteine) and either glucose (0.4%), arabinose (0.2%), or other specified carbon source. Cells were grown at 37°C or at 20°C until an early exponential phase (A_660_ =  ∼0.3) and pulse-labeled with 3.7 MBq/ml of [^35^S]methionine (obtained from PerkinElmer) for 0.5 min. Chase was initiated by adding unlabeled L-methionine to a final concentration of 200 µg/ml. At each sampling time, a 0.1 ml part of the culture was directly mixed with an equal volume of ice-cold 10% trichloroacetic acid. After standing on ice for 15 min or more, samples were micro-centrifuged at 4°C for 2 min and supernatant was discarded by aspiration. Precipitates were then vortexed with 1 ml of acetone, centrifuged again, and dissolved in SDS sample buffer (62.5 mM Tris-HCl pH 6.8, 2% SDS, 10% glycerol, 5% β-mercaptoethanol, a trace of bromophenol blue) described by Laemmli [32]. For most experiments, SDS sample buffer was treated with RNASecure (Ambion) according to the manufacturer's specification.

### Two-Dimensional Gel Electrophoresis for Detecting Cellular Nascent Polypeptides

Samples prepared as described above were usually mixed with pre-stained molecular weight marker, Precision Plus Blue (BioRad), and electrophoresed for the first dimension separation at 200 V for 35 min using 12% NuPAGE Bis-Tris gel and MES buffer available from Invitrogen [27]. Guided by the pre-stained markers, gel lanes were then cut out and incubated with 1 ml of 1 M Tris-base, containing 0.1% SDS, 10% glycerol and a trace of bromophenol blue, at 80°C for 20 min in a plastic bag. The gel piece was then placed on the top of the second dimension gel (12% NuPAGE Bis-Tris gel for 2D from Invitrogen) and overlaid with NuPAGE stacking gel solution (http://openwetware.org/wiki/Sauer:bis-Tris_SDS-PAGE%2C_the_very_best, except that acrylamide concentration was 5%) followed by its polymerization. We then carried out the second dimension electrophoresis (200 V for ∼35 min). The gel was dried and exposed to a phosphor imaging plate (Fuji Film), which was recorded by a BAS1800 or a FLA7000 imager (Fuji film). Quantification of the total gel-separated radioactivities and those on the nascent line was carried out by Image Gauge (Fuji Film) or Image Quant (GE health science) software to obtain %NL values.

### *In vitro* Translation of Truncated pro-*ompA* Messages

*In vitro* translation reaction using PURE System [31] and proOmpA_1-143_ truncated templates that end with an additional amino acid that altogether covers the 20 amino acids were described previously [5].

### Supporting Information

#### An earlier version of two-dimensional separation

We initially used incubation of the first dimension gel lanes at 70°C with 0.2 M Tris-base to hydrolyze the ester bonds of polypeptidyl-tRNAs. The gel lane was then placed on the top of a Laemmli gel [32] and subjected to the second dimension separation (Figure S1). *E. coli* proteins were pulse-labeled for 0.5 min at 20°C and examined by this separation system. Whereas simple polypeptides align along the main diagonal line (Figure S1A, completed chains; Figure S1C, after chase for 4 min), the pulse-labeled sample gave two additional, off-diagonal lines, one (“nascent line 1”) below and the other (“nascent line 2”) slightly above the main diagonal line. Consistent with their being nascent polypeptidyl-tRNA chains, the nascent lines 1 and 2 disappeared after treatment of the sample with RNase A before electrophoresis (Figure S1B) as well as after chase for 4 min (Figure S1C). Nascent line 1 should have contained molecules that were originally tRNA-linked and therefore migrating slower in the first dimension than in the second dimension.

We reason that pulse-labeled materials on nascent line 2 represented a class of polypeptidyl-tRNAs that were sensitive to RNase A but resistant to the in-gel hydrolysis conditions and that the NuPAGE and the Laemmli systems affected electrophoretic mobilities differentially between simple polypeptides and polypeptidyl-tRNAs. Indeed, nascent line 2 decreased only slowly during incubation at 70°C with 0.2M Tris-base (Figure S2A). The use of the same NuPAGE system for the second dimension electrophoresis led the nascent line 2 materials to completely merge into the main diagonal line (Figure S2B). These results raised the possibility that some species of polypeptidyl-tRNAs resisted the hydrolysis conditions we set up initially.

### Detection of a constitutively stalling SecM derivative on the nascent line

We detected a SecM variant with defective signal sequence ((ΔLGLPA)SecM), which undergoes constitutive elongation arrest [38], on the nascent line 1, when it was overproduced from a plasmid (Figure S3A; induced, no chase). The SecM spot remained on the nascent line even after a chase for 4 min at 37°C as an outstanding spot (Figure S3A, 4 min chase). Whereas neither the bulk polypeptidyl-tRNA nor (ΔLGLPA)SecM was visible when the gel was stained with coomassie brilliant blue (Figure S3B), the spot of (ΔLGLPA)SecM was visible with silver- and immuno-staining (Figure S3C and D). Thus, our method is capable of detecting regulatory nascent peptides when they exist in sufficient quantity. However, many of such polypeptides might be too minute in abundance and we have so far been unable to detect chromosomally encoded wild-type SecM.

### Single disruption of *ssrA* or *arfA* does not pronouncedly stabilize polypeptidyl-tRNAs

We pulse-labeled a single Δ*arfA* mutant and a single Δ*ssrA* mutant for 0.5 min and chased for 1 min at 37°C. Two-dimensional separation showed that neither of them exhibited the polypeptidyl-tRNA stabilization (Figure S4) to the extent that was observed with the double depletion strain (Figure 3). However, the Δ*ssrA* mutant contained a slightly higher proportion of radioactivity on the nascent line as compared with the wild-type cells or the Δ*arfA* mutant (Figure S4B).

## Supporting Information

Figure S1 Electrophoretic visualization of nascent polypeptides with incomplete cleavage of the resistant class of peptidyl-tRNA ester bonds. In these experiments, conditions for in-gel cleavage of the ester bonds were less stringent than those described in Materials and Methods; excised first dimension gels were incubated with 0.2 M (instead of 1 M) (tris(hydroxymethyl)aminomethane (Tris-base) at 70°C for 15 min (in stead of at 80°C for 20 min). In addition, the Laemmli system (14% separation–5% stacking gel) [32] was used for the second dimension electrophoresis. (A) Strain MC4100 [54] was pulse-labeled with [^35^S]methionine for 0.5 min at 20°C. Labeled macromolecules were separated by NuPAGE and subjected to in-gel incubation as described above and then to the second dimensional separation and phosphor imaging. Polypeptidyl-tRNAs form off-diagonal, two lines, “nascent line 1” below the main diagonal line and “nascent line 2” slightly above the main diagonal line. (B) The same sample was treated with RNase A before electrophoresis. (C) Pulse-labeled cells were chased for 4 min and then processed as in (A).
(TIFF)

Figure S2 Polypeptidyl-tRNAs with hydrolysis-refractory ester bonds form nascent line 2. (A) MC4100 cells were pulse-labeled with [^35^S]methionine for 0.5 min at 37°C. Gel lanes of the first dimension electrophoresis were incubated at 70°C with 0.2 M Tris-base for the indicated time periods before the second dimension separation. The results show that the materials on nascent line 2 were only slowly hydrolyzed. (B) The gel lane that had been incubated with 0.2 M Tirs-base at 70°C for 5 min was subjected to the second dimension separation using the same NuPAGE system as the first dimension electrophoresis. Now nascent line 2 perfectly overlapped the main diagonal line.
(TIFF)

Figure S3 Constitutively arresting SecM variant outstands among chemically minute polypeptidyl-tRNAs on the nascent line. (A) Strain NH336, which carried pSTD343 (*lacI* ^Q^) and pNH30 (encoding (ΔLGLPA)SecM-Met_6_ under the *lac* promoter control) [38], were grown on M9 medium supplemented with glycerol (0.2%), maltose (0.2%), amino acids (20 µg/ml each, other than methionine and cysteine), ampicillin (50 µg/ml) and chloramphenicol (20 µg/ml) at 37°C and induced for the *lac* transcription with IPTG (1 mM) and cyclic AMP (5 mM) as indicated at the top. Cells were pulse-labeled with [^35^S]methionine for 45 sec and chased with unlabeled methionine for 4 min as indicated. Radioactive proteins were separated by the earlier version of nascentome two-dimensional separation (see Figure S1). We believe that these hydrolysis conditions does not affect the conclusion on SecM, because SecM-tRNA, having glycine at the C-terminal end [27], is expected to be cleaved efficiently under these conditions (see the main text and Figure 1A). (B) and (C). The same uninduced and induced samples as in (A) were electrophoresed and successively stained with coomassie brilliant blue (B) and with silver (C). (D) The same uninduced and induced samples as in (A) were electrophoresed and subjected to anti-SecM immunoblotting [38]. Arrows indicate the spot of (LGLPA)SecM-Met_6_.
(TIFF)

Figure S4 Effects of a single *ssrA* or *arfA* mutation on stability of polypeptidyl-tRNAs. Cells of W3110 (wild-type), TA331 (Δ*ssrA*) and CH101 (Δ*arfA*) [21] were grown at 37°C and pulse-labeled with [^35^S]methionine for 0.5 min followed by chase for 1 min. Radioactive proteins were separated by the two-dimensional system.
(TIFF)
