# Supplementary figures and images for: Nascentome Analysis Uncovers Futile Protein Synthesis in Escherichia coli
Source: PLoS One. 2011 Dec 5;6(12):e28413. doi: 10.1371/journal.pone.0028413 (PMC3230602; doi:10.1371/journal.pone.0028413)

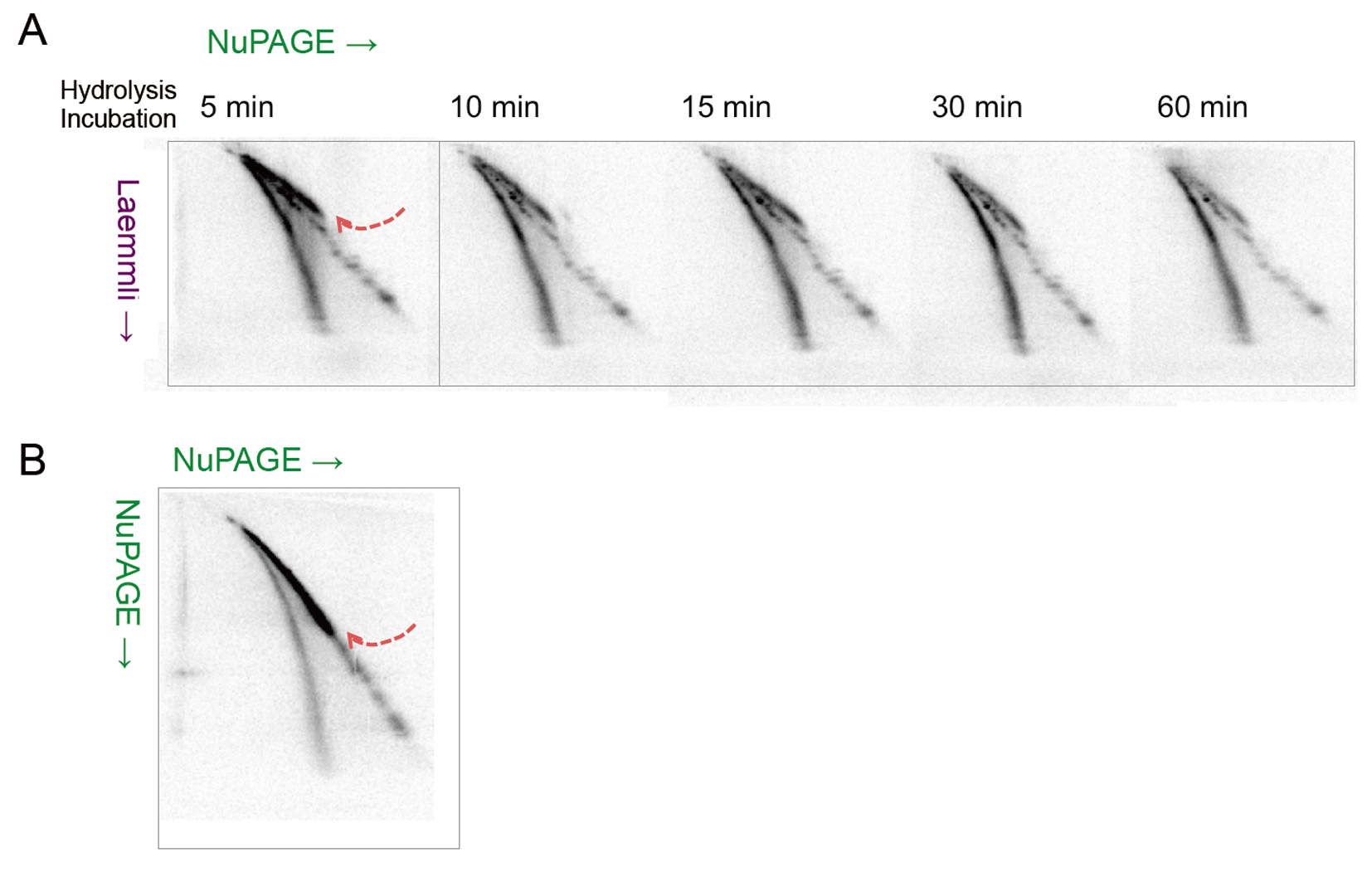

Supplement: Figure S2 — Polypeptidyl-tRNAs with hydrolysis-refractory ester bonds form nascent line 2. (A) MC4100 cells were pulse-labeled with [35S]methionine for 0.5 min at 37°C. Gel lanes of the first dimension electrophoresis were incubated at 70°C with 0.2 M Tris-base for the indicated time periods before the second dimension separation. The results show that the materials on nascent line 2 were only slowly hydrolyzed. (B) The gel lane that had been incubated with 0.2 M Tirs-base at 70°C for 5 min was subjected to the second dimension separation using the same NuPAGE system as the first dimension electrophoresis. Now nascent line 2 perfectly overlapped the main diagonal line. (TIFF) [file pone.0028413.s002.tiff]

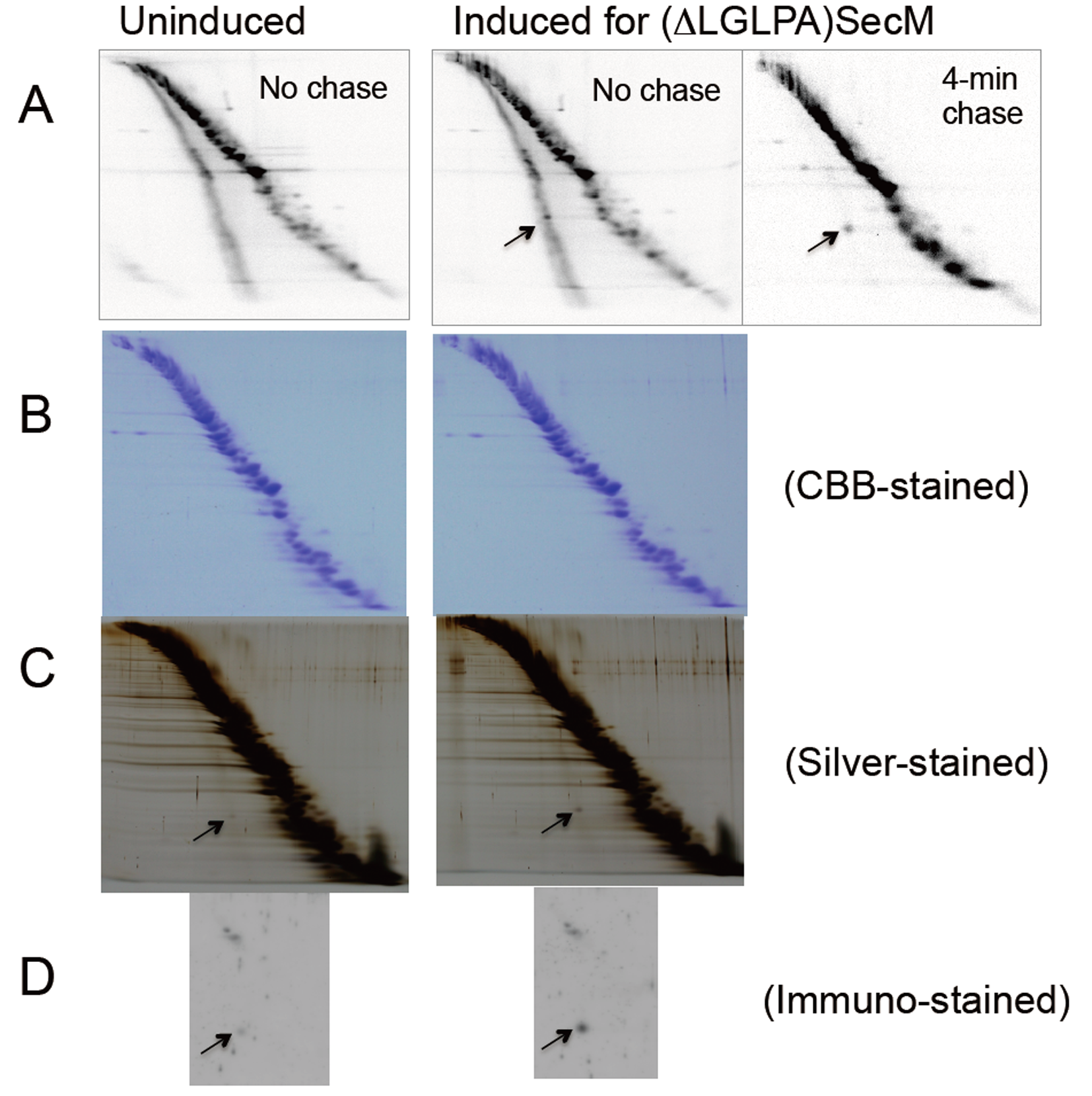

Supplement: Figure S3 — Constitutively arresting SecM variant outstands among chemically minute polypeptidyl-tRNAs on the nascent line. (A) Strain NH336, which carried pSTD343 (lacI Q) and pNH30 (encoding (ΔLGLPA)SecM-Met6 under the lac promoter control) [38], were grown on M9 medium supplemented with glycerol (0.2%), maltose (0.2%), amino acids (20 µg/ml each, other than methionine and cysteine), ampicillin (50 µg/ml) and chloramphenicol (20 µg/ml) at 37°C and induced for the lac transcription with IPTG (1 mM) and cyclic AMP (5 mM) as indicated at the top. Cells were pulse-labeled with [35S]methionine for 45 sec and chased with unlabeled methionine for 4 min as indicated. Radioactive proteins were separated by the earlier version of nascentome two-dimensional separation (see Figure S1). We believe that these hydrolysis conditions does not affect the conclusion on SecM, because SecM-tRNA, having glycine at the C-terminal end [27], is expected to be cleaved efficiently under these conditions (see the main text and Figure 1A). (B) and (C). The same uninduced and induced samples as in (A) were electrophoresed and successively stained with coomassie brilliant blue (B) and with silver (C). (D) The same uninduced and induced samples as in (A) were electrophoresed and subjected to anti-SecM immunoblotting [38]. Arrows indicate the spot of (LGLPA)SecM-Met6. (TIFF) [file pone.0028413.s003.tiff]
